# Refined detection and phasing of structural aberrations in pediatric acute lymphoblastic leukemia by linked-read whole-genome sequencing

**DOI:** 10.1038/s41598-020-59214-w

**Published:** 2020-02-13

**Authors:** Jessica Nordlund, Yanara Marincevic-Zuniga, Lucia Cavelier, Amanda Raine, Tom Martin, Anders Lundmark, Jonas Abrahamsson, Ulrika Norén-Nyström, Gudmar Lönnerholm, Ann-Christine Syvänen

**Affiliations:** 1 Department of Medical Sciences, Molecular Medicine and Science for Life Laboratory, Uppsala University, Uppsala, Sweden; 2 Department of Immunology, Genetics and Pathology and Science for Life Laboratory, Uppsala University, Uppsala, Sweden; 3 Department of Pediatrics, Institution for Clinical Sciences, Sahlgrenska Academy, Gothenburg University, Gothenburg, Sweden; 4 Department of Clinical Sciences and Pediatrics, University of Umeå, Umeå, Sweden; 5 Department of Women’s and Children’s Health, Pediatric Oncology, Uppsala University, Uppsala, Sweden

**Keywords:** Molecular medicine, Cancer genomics, Acute lymphocytic leukaemia

## Abstract

Structural chromosomal rearrangements that can lead to in-frame gene-fusions are a leading source of information for diagnosis, risk stratification, and prognosis in pediatric acute lymphoblastic leukemia (ALL). Traditional methods such as karyotyping and FISH struggle to accurately identify and phase such large-scale chromosomal aberrations in ALL genomes. We therefore evaluated linked-read WGS for detecting chromosomal rearrangements in primary samples of from 12 patients diagnosed with ALL. We assessed the effect of input DNA quality on phased haplotype block size and the detectability of copy number aberrations and structural variants in the ALL genomes. We found that biobanked DNA isolated by standard column-based extraction methods was sufficient to detect chromosomal rearrangements even at low 10x sequencing coverage. Linked-read WGS enabled precise, allele-specific, digital karyotyping at a base-pair resolution for a wide range of structural variants including complex rearrangements and aneuploidy assessment. With use of haplotype information from the linked-reads, we also identified previously unknown structural variants, such as a compound heterozygous deletion of *ERG* in a patient with the *DUX4*-*IGH* fusion gene. We conclude that linked-read WGS allows detection of important pathogenic variants in ALL genomes at a resolution beyond that of traditional karyotyping and FISH.

## Introduction

Sequencing of complete human genomes has become feasible owing to next generation sequencing (NGS) technologies, but detection of the whole spectrum of somatic single nucleotide variants (SNVs), copy number alterations (CNAs), and structural variations (SVs) in cancer cells remains challenging^1^. The human genome is diploid, and molecular haplotyping of the two alleles across large genomic regions is beyond the resolution of standard short-read NGS technologies^2^. “Linked-read” technology, by which single DNA molecules are massively barcoded in a microfluidic format and subsequently sequenced using short-read NGS technology, allows determination of molecular haplotypes across mega-base regions of the genome^3–5^. An advantage of linked-read whole genome sequencing (WGS) is its enhanced ability to detect the breakpoints of SVs and to provide long-range haplotype information for phasing SNVs and SVs. Linked-read WGS has the potential to provide an ordered view of the structure of all genetic variants in a genome, shown by assignment of complex SVs, chromosomal rearrangements, and CNAs to individual chromosomes in germline and cancer genomes^3,5,6^.

Structural chromosomal rearrangements that may lead to aberrant gene-fusions are used for diagnosis, risk stratification and prognosis in pediatric acute lymphoblastic leukemia (ALL)^7^. Several recurrent chromosomal aberrations define genetic subtypes of ALL that are associated with clinical outcome^8,9^. Karyotyping (G-banding) and fluorescent *in situ* hybridization (FISH) commonly applied in clinical genetics laboratories do not capture the full spectrum of complex aberrations in cancer genomes. Thus, up to 30% of B-cell precursor ALL (BCP-ALL) patients remain cytogenetically unclassified and lack genetic information as support for treatment decisions^10^. More recently, the application of WGS and whole-transcriptome sequencing (RNA-sequencing) have enabled discovery of novel mutations and expressed gene-fusions in ALL^11–16^ including recurrent fusion genes with biological and clinical implications, such as *DUX4, ZNF384*, and *MEF2D* rearrangements^17–19^. However, limited information presently exists on the complex structure of the leukemogenic aberrations present in ALL genomes.

Here, we use linked-read WGS technology to obtain haplotype-resolved genomic aberrations in primary DNA samples from 12 well-characterized patients with pediatric ALL. Furthermore, we evaluate if linked-read WGS can achieve the same or improved level of detection as joint G-banding and FISH.

## Results

We subjected diagnostic samples from 12 children with acute lymphoblastic leukemia (ALL) enrolled on the Nordic Society of Pediatric Hematology and Oncology (NOPHO) protocols during 1998–2008 (Table 1)^8,20^ to linked-read WGS (Table S1). The DNA used to prepare linked-read sequencing libraries was obtained from biobanked DNA isolated by a standard column-based method or by freshly prepared HMW DNA extraction. The estimated length of the input DNA was directly correlated to the phase block size (Table S2). The proportion of phased SNPs was 81–99% (mean 92%), and the longest phased blocks ranged from 0.9–18 Mb (mean 7 Mb) (Table S3). The DNA extracted using the High Molecular Weight protocol yielded the longest haplotype blocks (18 Mb), but the DNA extracted by the standard column-based method allowed for detection of all known SVs even at low sequencing coverage (10×), despite the shorter phase blocks produced (Fig. S1).

**Table 1:** Patient characteristics.

Patient ID	Sex	Age at diagnosis	Immuno-phenotype	Subtype at diagnosis	Revised subtype	Karyotype at diagnosis	Revised karyotype after linked-read WGS
ALL_370	F	3	BCP-ALL	HeH	—	55, XX, +X, +4, +6, +10, +14, +17, +18, +21, +21[2]/54, XX, +X, +4, +6, +10, i(14)(q10), +17, +18, +21, +21[cp16]/46, XX[12]	55, XX, +X, +4, +6, +10, +14, +17, +18, +21, +21
ALL_689	F	18	BCP-ALL	HeH	—	55, XX, +X, dup(1)(q24q32), +4, +6, +10, +14, +17, +18, +21, +21[17]/46, XX[3]	**54**, XX, +X, dup(1)(q24**q42**), +4, +6, +10, +14, +17, +18, +21
ALL_47	M	2	BCP-ALL	Normal karyotype	HeH	46, XY[2]	**58, XY, +4, +5, +6, +9, +10, +12, +14, +17, +18, -19, +19, +21, +21, +22**
ALL_458	M	4	BCP-ALL	*ETV6-RUNX1*	—	.ish.t(12;21)(p13;q22), del(12)(p13p13), del(21)(q22q22)	**47**, XY**, +10, del(11)(q22.1q25)**, t(12;21)(p13.2;q22), del(12)**(p12.1p13.2)**
ALL_386	M	13	BCP-ALL	*ETV6-RUNX1*	—	.ish.t(3;21;12), t(3;12;14), t(12;21)(p13;q22)	**46, XY, del(2)(q33.1q37.3), der(3)del(3)(p21.2p21.31)t(3;12)(p21.31;q24)ins(3;3)(q21.2;p21.31p21.31), der(12)t(14;12)(q24.1;p13.2)t(3;12)(q21.3;q24.11), del(12)(p13.2), der(14)t(14;2)(q24.1;q37.3)t(2;21)(q33.1;q22.12), del(19)(q13.32q13.43), der(21)t(12;21)(p13.2;q22.12), dup(21)(q11.2q22.12)**
ALL_402	M	6	BCP-ALL	*BCR-ABL1*	—	46, XY[12].ish.t(9;22)(q34;q11), del(9)(p21p21)	46, XY, t(**1;5**;9;22)(**p36.33;q31.2**;q34.12;q11.23), **del(8)(p11p23), dup(8)(p11.23q24.3)**, del(9)(p21p21)
ALL_390	F	8	BCP-ALL	Normal karyotype	*DUX4-IGH*	46, XX[19]	46, XX, **del(6)(q14.1q27)**
ALL_501	F	7	BCP-ALL	Normal karyotype	*DUX4-IGH*	46, XX[20]	46, XX
ALL_604	M	11	BCP-ALL	B-other	*TCF3-ZNF384*	46, XY, del(7)(q22)[8]/46, XY, del(6)(q2?1)[7]/ 46, XY[17]	46, XY, del(6)**(q16.2q22.33)**, del(7)**(q21.3q36.3), t(12;19)(p13.31;p13.3)**
ALL_613	M	5	BCP-ALL	B-other	*EP300-ZNF384*	46, XY, del(16)(q13q24)[5]/47-48, XY, +del(1)(q21), del(16)(q13q24), +mar[cp3]/ 46, XY[9]	46, XY, **dup(1)(q21q44)**, **t(12;22)(p13.2;q13.2)**, del(16)(**q21**q24.3)
ALL_707	M	2	BCP-ALL	B-other	*PAX5-ELN*	46, XY, der(7)t(7;9)(q11;p13)del(9)(p21p24), der(9)t(7;9)(q11;p13)[9]/46, XY, idem, del(19)(q13)[15]/46, XY[1]	46, XY, **del(7)(q11)**, der(9)t(7;9)(q11;p13), del(9)(**p13**p24)
ALL_559	M	6	T-ALL	T-ALL	—	46, XY, t(7;9)(q3?4;q3?2)[10].ish.del(9)(p21p21)x2, der(11)t(7;11)(q3?4;p1?3)/46, XY[15]	46, XY, **der(7)t(7;9)(q34;q31), t(7;11)(q34;p15), der(9)t(7;9)(q34;q31)**del(9)(p21p21), del(9)(p21p21)

^a^The parts of the karyotype revised after linked-read WGS are highlighted in bold.

For five of the 12 ALL genomes, detailed karyotype information obtained at diagnosis by G-banding or FISH for the subtype-defining genetic aberrations high hyperdiploidy (HeH), t(12;21) and t(9;22) was available and allowed verification of the results from linked-read WGS. The remaining six patients with either T-ALL or B-other subtype had either complex or incomplete karyotype available from ALL diagnosis. Their subtypes were determined in previous studies by a combination of WGS, RNA-sequencing, and/or arrays (Table S1)^11,19^. In all cases, existing karyotype information, newly generated FISH data (when cells were available), and/or a combination of Infinium arrays for copy number estimates and RNA-sequencing validated the findings from linked-read WGS. The results for each patient and subtype are detailed below, and for each case a revised karyotype after linked-read WGS is given in Table 1.

### High Hyperdiploidy (HeH)

**Figure 1:**
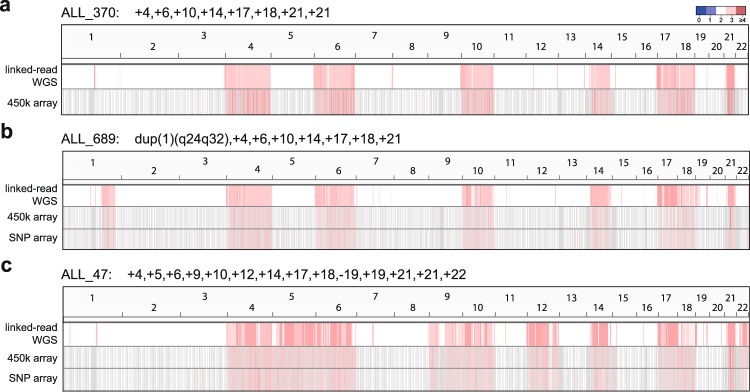
Copy number by chromosome for the three ALL patients with the HeH subtype (**a–c**). The average linked-read WGS coverage calculated in 10 Kb bins is plotted in the top row of each panel. The Log R ratios from Infinium SNP and/or 450k array data are visualized in the lower part of each panel. Red coloring indicates chromosomal gains according to the color key above panel a.

Two patients (ALL_370 and ALL_689) had the classical HeH subtype with 55 chromosomes. Using the linked-read WGS data, we binned the average sequencing coverage in 10 Kb bins across the genome and scanned for CNAs across the 22 autosomal chromosomes (Fig. 1a,b). The linked-read WGS estimates of copy numbers correlated perfectly with that from the karyotypes and array-based CNA for ALL_370 and ALL_689. For a third patient (ALL_47) with suspected HeH subtype^21^, we verified the HeH karyotype in the linked-read WGS data to be 58, XY, +4, +5, +6, +9, +10, +12, +14, +17, +18, −19, +19, +21, +21, +22, which was confirmed by array-based CNA analysis (Table 1; Fig. 1c). The copy neutral loss of chromosome 19 (uniparental disomy) was visible in the linked-read WGS data by an overrepresentation of homozygous SNVs on chromosome 19 (Fig. S2).

### Translocations t(12;21) and t(9;22)

**Figure 2:**
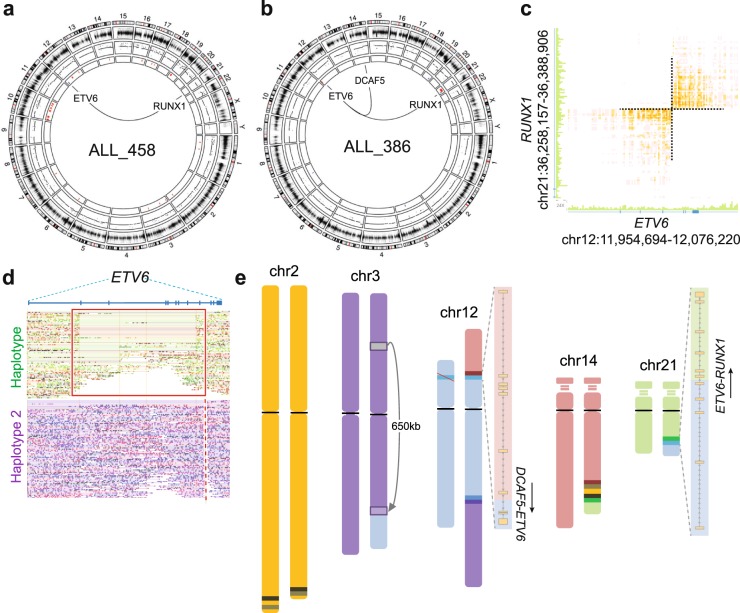
Structural aberrations detected by linked-read WGS in t(12;21)[*ETV6*-*RUNX1*] genomes. (**a,b**) Circos plots for patients ALL_386 and ALL_458. The first (outer) track shows the chromosomes and their banding, the second track shows log R ratios from Infinium arrays, the third track shows copy number determined by linked-read WGS in 10 Kb bins, and the fourth (innermost) track shows copy number calls using the CNVnator software. Red indicates gain and blue indicates deletion. Expressed fusion genes are highlighted within each circos plot, solid lines indicate in-frame fusion genes. (**c**) Heatmap of overlapping linked-reads supporting a balanced inter-chromosomal translocation t(12;21) resulting in the *ETV6-RUNX1* fusion gene in ALL_486. (**d**) Linked-reads mapped to the two haplotypes at the *ETV6* locus in patient ALL_386, which depicts a deletion on haplotype 1 (indicated by the red box) and the breakpoint giving rise to the *DCAF5*-*ETV6* and the *ETV6*-*RUNX1* fusion genes is indicated by a dashed line on the second allele (haplotype 2). (**e**) Schematic representation of the chromosomal rearrangements resulting in derivative chromosomes as determined by linked-read WGS in ALL_386. The resulting fusion transcripts with breakpoints are drawn alongside the chromosomes involved in the translocations.

The t(12;21)[*ETV6-RUNX1*] translocation and associated aberrations were determined in two patients (ALL_386 and ALL_458) (Fig. 2a,b). As anticipated by karyotyping and previous WGS of patient ALL_458^11^, a balanced t(12;21) translocation resulting in the expression of both the canonical *ETV6-RUNX1* and the reciprocal *RUNX1-ETV6* fusion genes was unambiguously detected at base-pair resolution in the linked-read WGS data (Fig. 2c). A deletion spanning over a 2.1 Mb region that includes the second allele of *ETV6* was observed on the other haplotype, thus affecting both alleles of *ETV6* (Fig. S3**)**. Besides gain of chromosome 10 and a heterozygous 38 Mb deletion of chromosome 11q22-q25, no other large structural variants were identified in ALL_458.

In contrast, the karyotype for patient ALL_386 suggested a complex series of translocations involving *ETV6* and *RUNX1* and chromosomes 3, 12, 14 and 21. In a previous study, two in-frame fusion genes were identified in this patient (*ETV6-RUNX1* and *DCAF5-ETV6*)^19^. Linked-read data resolved that the *DCAF5-ETV6* fusion gene arose from a translocation between 14q24.1 and 12p13.2 and the *ETV6*-*RUNX1* fusion gene arose from a translocation between 12p13.2 and 21q22.12. The phasing information further resolved a heterozygous 0.15 Mb intragenic deletion in *ETV6* (haplotype 1) and that the *ETV6-RUNX1* and *DCAF5-ETV6* fusion genes originated from the other allele (haplotype 2) of *ETV6*, thus disrupting both copies of *ETV6* in this patient (Fig. 2d). Linked-read WGS resolved the exact breakpoints on chromosomes 3, 12 14 and 21, and identified several additional alterations that were missed by genetic analysis at diagnosis. Of these, *DCAF5* (chr14) and the reciprocal *RUNX1* (chr21) loci were separated by a 44 Mb insertion of a region originating from chromosome 2q33.1-q37.3 on the derivative chromosome 14q24.1 (Fig. 2e). Furthermore, a 650 Kb region from chromosome 3p21.31 was inverted and inserted into the derivative chromosome 3q21.2 arm where the material from chromosome 12q24.13 was translocated (Fig. S4). All of the derived chromosomes determined by linked-read WGS were subsequently validated by FISH (Fig. S5).

**Figure 3:**
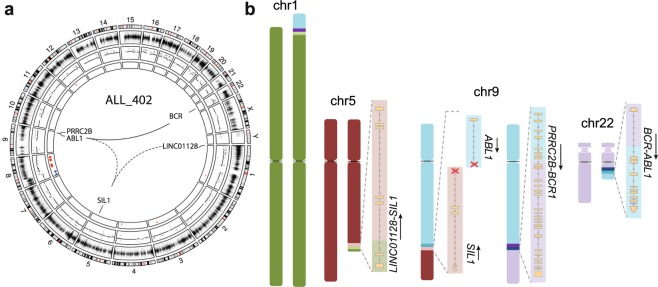
Complex structural rearrangements in the patient ALL_402. (**a**) A circos plot depicting the genome-wide copy number changes in ALL_402. The first (outer) track shows each chromosome and their banding, the second track shows log R ratios from infinium arrays, the third track shows copy number determined by linked-read WGS in 10 Kb bins, and the fourth (innermost) track shows copy number calls using the CNVnator software. Red indicates gain and blue indicates deletion. Expressed fusion genes are highlighted inside of the circos plot, solid lines indicate in-frame and dashed lines indicate out of frame fusion or truncated genes. (**b**) The derivative chromosomes as outlined using linked-read WGS. The structures of the expressed fusion genes are shown alongside their derivative chromosomes with the direction of transcription indicated by arrows.

In patient ALL_402 with t(9;22)[*BCR-ABL1*], linked-read WGS revealed an unexpectedly complex rearrangement that involved the *BCR* (22q11.23), *ABL1* (9q34.12), *PRRC2B* (9q34.13), *SIL1* (5q31.2) and *LINC01128* (1p36.33) loci (Fig. S6). In addition to the deletion of chromosome 9p21 reported in the karyotype, we detected a 35 Mb deletion (8p11.23-p23.3) and a gain starting at 8p11.23 and continuing through the entire q-arm of chromosome 8 (Fig. 3a). RNA-sequencing verified that the 5′ end of *BCR* is fused with the 3′ end of *ABL1*, the 5′ ends of the reciprocal *ABL1* and *SIL1* loci form a head to head translocation, resulting in two truncated transcripts, the 5′ end of *LINC01128* is fused with the 3′ end of *SIL1*, whilst the 5′ end of *PRRC2B* is fused with the reciprocal 3′ end of the *BCR* gene (Fig. 3b). None of these complex rearrangements were phased in the linked-read WGS data, but phasing information was not required to fully resolve the structure of the breakpoints in this case.

### B-other group

**Figure 4:**
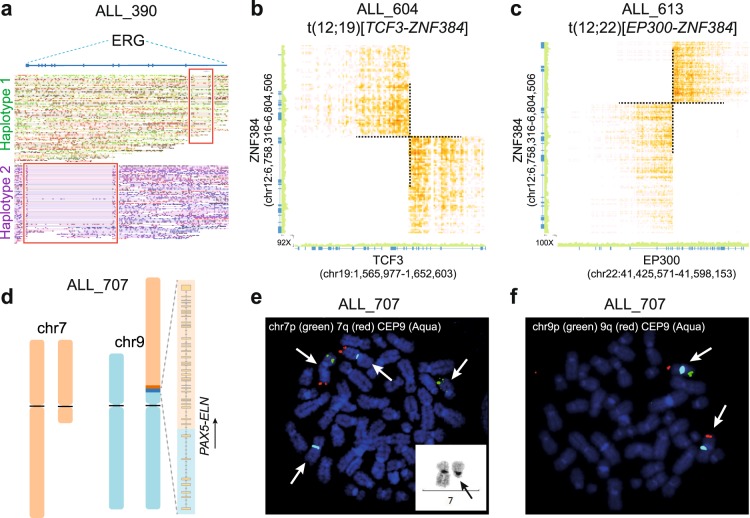
Structural rearrangements detected in B-other patients by linked-read WGS. (**a**) Linked-reads mapped to each of the two homologous chromosomes at the *ERG* locus on chromosome 21 in patient ALL_390. Reads are color-coded by chromosome and deletions are marked by red squares. B-C) Heatmaps of overlapping linked-reads supporting subtype-defining balanced inter-chromosomal translocations from the 10x Genomics Loupe software. (**b**) The genomic breakpoint in chromosomes 12 and 19, resulting in the *TCF3-ZNF384* fusion gene in patient ALL_604. (**c**) The genomic breakpoint in chromosomes 12 and 22, resulting in the *EP300-ZNF384* fusion gene in patient ALL_613. **(d**) Ideogram of the structure of the translocation between chromosome 7 and 9 in the patient ALL_707 resulting in the *PAX5-ELN* fusion gene, which is shown besides the derivative chromosome 9 with the direction of the transcription indicated by an arrow. (**e,f**) Validation of the chromosome 7q deletion and derivative chromosome 9 by FISH in the patient ALL_707.

*DUX4* and *ZNF384*-rearrangements define newly described subtypes of BCP-ALL that were initially detected in large-scale RNA-sequencing studies^17,18,22^. The *DUX4-IGH* fusion gene results from an insertion of the *DUX4* gene (subtelomeric region of chr4q and 10q), into the enhancer region of the *IGH* locus (chr14)^23^. With the exception of a 93 Mb deletion on chromosome 6q14.1-q27 in ALL_390, the two patients with *DUX4-IGH* (ALL_390 and ALL_501) had normal karyotypes typical of this subtype (Fig. S7). Previous short-read WGS of ALL_501 failed to identify the *DUX4-IGH* rearrangement in this patient^11^. The *DUX4-IGH* rearrangement was not directly detected in the linked-read data by the longranger software, however with the aid of the Integrated Genome Viewer, we were able to identify split linked-reads supporting the insertion of at least one copy of *DUX4* into the *IGH* locus, thus supporting the rearrangement (Fig. S8). Besides the 6q deletion in ALL_390, the linked-read data revealed a compound heterozygous deletion of *ERG* transcript variant 1 (NCBI Reference Sequence: NM_182918.3). A large 6.5 Mb phase block on chromosome 21q22 enabled detection of a 9.3 Kb focal deletion of exon 1 on haplotype 1 and a separate 57.2 Kb deletion spanning exons 3–10 on haplotype 2 (Fig. 4a).

The most common fusion gene partners of *ZNF384* are the *TCF3* and *EP300* genes. Linked-read WGS determined the chromosomal breakpoints at base-pair resolution for the balanced translocations t(12;19)(p13.31;p13.3)[*TCF3-ZNF384*] in ALL_604 and t(12;22)(p13.31;q13.2)[*EP300-ZNF384*] in ALL_613 (Fig. 4b,c). The heterozygous deletions expected from the karyotypes in ALL_604 and ALL_613 were refined by linked-read WGS to 7q21.3-q36.3 and 6q16.2-q22.33 in ALL_604, and 16q21-q24.3 in ALL_613 (Table 1; Fig. S9). Gain of the q arm of chromosome 1, a common genomic aneuploidy in ALL^24^ was observed in the linked-read data from patient ALL_689, but not in the diagnostic karyotype.

One patient with a *PAX5*-*ELN* fusion gene (ALL_707) detected by RNA-sequencing and short-read WGS was included^11^. The karyotype indicated two derivative chromosomes (chromosome 7 and 9) as well as a 9p deletion. These aberrations were resolved at a higher resolution with linked-read WGS, which demonstrated a derivative chromosome 9 harboring the *PAX5*-*ELN* fusion gene, a truncated chromosome 7, as well as a heterozygous deletion of chromosome 9p13.2 with the breakpoint in the *PAX5* locus (Fig. S10). The structure of the resulting derivative chromosomes and their validation by FISH are shown in Fig. 4d–f.

### T-ALL

**Figure 5:**
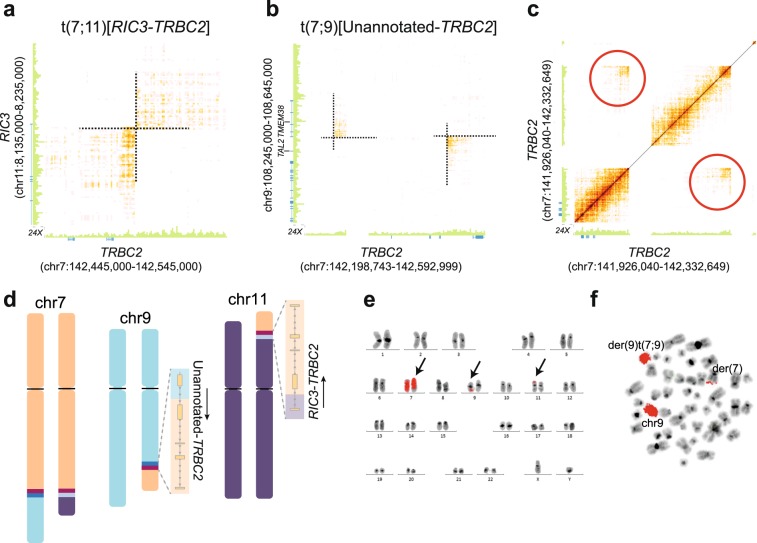
Chromosomal aberrations in the patient ALL_559 (T-ALL) determined by linked-read WGS. (**a–c**) Heatmaps from the 10x Genomics Loupe software of overlapping linked-reads indicating genomic rearrangements. (**a**) A balanced interchromosomal translocation between chromosomes 7 and 11. (**b**) A translocation between chromosomes 7 and 9, which is accompanied by a 0.2 Mb deletion flanked by an inversion of chromosome 7q34 on the second allele at the *TRBC2* locus. The translocation results in an expressed fusion gene between *TRBC2* and an unannotated gene located 500 bp upstream of *TMEM38B* on chromosome 9. (**c**) Zoomed in view of the inversion flanking the *TRBC2* locus on 7q34. (**d**) Ideogram of the structure of the translocations observed in ALL_559. The chromosomes are drawn to scale using the CyDAS software. (**e**) Whole chromosomal paint depicting the translocation of material from chromosome 7 to chromosomes 9 and 11. (**f**) Whole chromosomal paint of chromosome 9 depicting the balanced translocation involving chromosome 7.

Based on karyotype, a bi-allelic deletion of chromosome 9p21 and two translocations involving chromosomes 7 and 9 and chromosomes 7 and 11 were expected in ALL_559. The homozygous deletion of chromosome 9p21 was clearly resolved in the linked-read WGS data **(**Fig. S11**)**. Previous short-read WGS and RNA-sequencing data identified two translocations involving the T-cell receptor beta locus (*TRBC2* gene) on chromosome 7, namely t(7;11)(q34;p15)[*RIC3-TRBC2*] and t(7;9)(q34;q31) resulting in the fusion of *TRBC2* with an unannotated transcript expressed on chromosome 9 between the *TAL2* and *TMEM38B* genes^11^. The linked-read WGS data clarified that the two alleles of *TRBC2* were involved in independent translocation events. First, the t(7;11)(q34;p15) resulting in expression of *RIC3-TRBC2* was a consequence of a balanced translocation of chromosome 7 involving one allele of *TRBC2* (Fig. 5a). On the other allele of *TRBC2*, the t(7;9)(q34;q31) was accompanied by a 0.2 Mb deletion flanked by an inversion of chromosome 7q34 (Fig. 5b–d), a re-arrangement that was missed by both karyotyping and previous short-read WGS^11^. FISH verified the derivative chromosomes determined by linked-read WGS (Fig. 5e,f).

### Detection of key diagnostic deletions for ALL

To further demonstrate that linked-read WGS allows detection of other aberrations than large-scale aneuploidies and translocations, we screened the 12 ALL genomes for focal deletions in a set of relevant genes for ALL, including *BTG1*, *CDKN2A/B*, *EBF1*, *ETV6*, *IKZF1*, *PAX5*, *RB1 and ERG*^25^ (Fig. S12). With the exception of RB1, each of the genes analyzed were deleted in at least one patient based on linked-read WGS. All deletions were verified by array-based CNA analysis. Phasing data revealed that both of the t(12;21) cases harbored *ETV6* deletions on the allele that was not affected by the translocation, thus resulting in bi-allelic disruption of *ETV6*. Consistent with previous studies^23,26^, recurrent *BTG1* and *IKZF1* deletions were detected in the t(12;21) and *DUX4-IGH* patients, respectively (Fig. S13).

## Discussion

In our study the linked-reads enabled highly accurate resolution of the majority of the genomic aberrations defined by cytogenetic methods and refined or identified new structural rearrangements in 10 of the 12 analyzed ALL genomes. Although the ALL subtypes and numbers of samples were modest, these results show clear proof of principle for linked-read WGS for digital karyotyping in ALL. Studies that have applied linked-read-WGS to other cancer types such as triple negative breast cancer^27^, metastatic gastric tumors^28^, prostate cancer^29^, and cell lines^30,31^ have reached similar conclusions.

Linked-read WGS requires long input DNA molecules to gain the most benefit from the technology^3^. However, when working with clinical samples, high molecular weight DNA extraction and handling of HMW DNA is not practical in most clinical settings. In our study we showed that DNA from patient samples with an average size of DNA < 50 kb prepared using a standard column-based DNA extraction method were highly informative for detection of genomic aberrations with linked-read WGS. When we compared HMW DNA to DNA from standard column extractions, and when we compared low-coverage GemCode to Chromium library preparation, the results were concordant. Although HMW DNA may increase the chances of phasing over chromosomal breakpoints, which makes interpretation of the chromosomal structure and organization easier, our data suggest that long DNA molecules and high sequencing depth may not be required for accurate detection of prognostically relevant aberrations present in the major clone of leukemic samples.

Although the genomic structure of most chromosomal rearrangements that are of clinical relevance in ALL were resolved with high precision by linked-read WGS, the recently described *DUX4*-*IGH* fusion gene failed to be precisely resolved by this technology. The *DUX4*-*IGH* rearrangement is a particularly challenging aberration to resolve due to the location of *DUX4* in the complex tandemly repeated region D4Z4 in the subtelomeric region of chr4q and chr10q^32^, and the insertion of *DUX4* into the *IGH* locus. This complexity is likely the reason for the lack of identification of the recurrent *DUX4-IGH* fusion gene prior to recent RNA-sequencing studies in ALL^17–19^. Nonetheless, a guided analysis based on identifying split linked-reads that map to the *DUX4* and *IGH* loci identified support for the insertion of at least one copy of *DUX4* into the *IGH* locus in the linked-read WGS data.

The present study is limited by the fact that we have not compared the linked-reads to other next generation approaches such as standard paired-end WGS, Hi-C, third-generation single-molecule sequencing, or optical mapping technologies, which when used in a multiplatform approach have been demonstrated to be a powerful method for resolving complex structural rearrangements^33–35^. Future studies will be required for more formal benchmarking of linked-read WGS and other next generation technologies for digital karyotyping specifically in ALL and for other cancer types.

In summary, we focused on detecting large-scale structural aberrations, which are the most relevant type of aberrations for clinical care in ALL^36^. We generated a detailed view of large-scale chromosomal aberrations in cells from pediatric ALL patients, which reaches beyond the resolution of traditional karyotyping data^11,12,37^. Our data suggests that digital karyotyping by linked-read WGS can replace, or at the least complement traditional clinical diagnostic methods such as G-banding and FISH in the future.

## Patients and Methods

### Patient samples

Primary ALL samples were collected as described previously^38^. The patients were selected from the NOPHO cohort based on presence of cytogenetic aberrations detected at diagnosis or fusion genes detected by previous WGS or RNA-sequencing studies **(**Table S1**)**^11,19,21^. DNA and RNA were extracted from 2–10 million cells using the AllPrep DNA/RNA Mini Kit, AllPrep DNA/RNA/miRNA Universal Kit, or the MagAttract HMW DNA kit (Qiagen). The DNA concentrations were measured using the Qubit dsDNA Broad Range assay (Invitrogen). The study was approved by the Regional Ethics Review Board in Uppsala, Sweden and was conducted according to the guidelines of the Declaration of Helsinki. The patients and/or their guardians provided written informed consent.

### Molecular diagnosis, karyotyping, and FISH

ALL diagnosis was established by analysis of leukemic cells with respect to morphology, immunophenotype, and cytogenetic aberrations. High hyperdiploidy (HeH) was defined as presence of 51–67 chromosomes per cell^39^. FISH or RT-PCR analyses were used to screen for t(12;21)(p13;q22)[*ETV6*-*RUNX1*] and t(9;22)(q34;q11)[*BCR*-*ABL1*]. Whole-chromosome paint (Metasystems XCP orange/green XCyting Chromosome Paints) and subtelomeric probes (Vysis Totelvysion probes) followed by analysis using a fluorescence microscope (Carl Zeiss) and the Isis software (MetaSystems) were used to validate translocations identified by linked-read WGS on metaphase spreads from cultured bone marrow cells.

### Library construction and sequencing

GemCode and Chromium libraries for linked-read WGS (10x Genomics) were prepared from 1–1.2 ng of genomic DNA following manufacturer’s protocols for GemCode and Chromium V1 reagents. GemCode libraries (n = 12) were sequenced on an Illumina HiSeq 2500 instrument (read1:98 bp, i7:8 bp, i5:14 bp, read2:98) to an average depth of 14×. Chromium libraries (n = 5) were sequenced on an Illumina HiSeqX instrument with 150 bp paired-end reads to an average depth of 32×.

### Linked-read data analysis

Linked-read WGS data was processed and phased using the Long Ranger pipeline from 10x Genomics (v1.2.0 for GemCode and v2.1.6 for Chromium) with the hg19/GRCh37 reference genome. Data were visualized using the Loupe Genome Browser v2.1.1. SVs called by Long Ranger were manually reviewed against karyotype data, CNA data from Illumina Infinium arrays, and fusion genes detected by RNA-sequencing. Genomic copy number levels were estimated by chromosomal segmentation read-depth analysis in 10 Kb windows using the CNVnator software^40^. B-allele frequencies were calculated from VCF files using the VariantAnnotation package and custom scripts in R^41^. Ideograms of derivative chromosomes were drawn to scale with the CyDAS software^42^.

### RNA-sequencing

A RNA-sequencing library was constructed from 300 ng total RNA with the TruSeq stranded total RNA protocol (Illumina) for sample ALL_402. The library was sequenced on a NovaSeq. 6000 instrument with 100 bp paired-end reads. Strand-specific RNA-sequencing data was available from previous studies for all of the remaining patient samples, except from patient ALL_370 where RNA was not available (Table S1)^11,19,21^. Fusion genes were called with FusionCatcher V0.99.7d^43^ and validated using a previously described approach^19^.

### Copy Number Analysis

Infinium HumanMethylation450 BeadChip (450k array) data from all samples are available at the Gene Expression Omnibus (GSE49031)^44^. The R package “CopyNumber450kCancer” was used to detect CNAs^45^. Genomic DNA (200 ng) from nine patient samples was genotyped on the Illumina HumanOmni2.5 Exome-8v1 SNP arrays (Illumina). CNAs were called from the SNP array data using the Tumor Aberration Prediction Suite^46^.

## Supplementary information

Supplementary Figures S1-S13.

Supplementary Tables S1, S2, S3.

## Data Availability

The copy number data generated in this study have been deposited in NCBI’s Gene Expression Omnibus and are accessible through GEO Series accession number GSE116057. The patient/parent consent does not cover depositing data that may be used for large-scale determination of germline variants in a repository.

## References

[1] Sheikine Y, Kuo FC, Lindeman NI (2017). Clinical and Technical Aspects of Genomic Diagnostics for Precision Oncology. Journal of clinical oncology: official journal of the American Society of Clinical Oncology.

[2] Porubsky D (2017). Dense and accurate whole-chromosome haplotyping of individual genomes. Nat Commun.

[3] Zheng GX (2016). Haplotyping germline and cancer genomes with high-throughput linked-read sequencing. Nature biotechnology.

[4] Weisenfeld NI, Kumar V, Shah P, Church DM, Jaffe DB (2017). Direct determination of diploid genome sequences. Genome research.

[5] Marks P (2019). Resolving the full spectrum of human genome variation using Linked-Reads. Genome research.

[6] Mostovoy Y (2016). A hybrid approach for de novo human genome sequence assembly and phasing. Nature methods.

[7] Iacobucci Ilaria, Mullighan Charles G. (2017). Genetic Basis of Acute Lymphoblastic Leukemia. Journal of Clinical Oncology.

[8] Schmiegelow K (2010). Long-term results of NOPHO ALL-92 and ALL-2000 studies of childhood acute lymphoblastic leukemia. Leukemia.

[9] Moorman AV (2012). The clinical relevance of chromosomal and genomic abnormalities in B-cell precursor acute lymphoblastic leukaemia. Blood reviews.

[10] Pui CH (2015). Childhood Acute Lymphoblastic Leukemia: Progress Through Collaboration. Journal of Clinical Oncology.

[11] Lindqvist CM (2015). The mutational landscape in pediatric acute lymphoblastic leukemia deciphered by whole genome sequencing. Human mutation.

[12] Holmfeldt L (2013). The genomic landscape of hypodiploid acute lymphoblastic leukemia. Nature genetics.

[13] Schwab C, Harrison CJ (2018). Advances in B-cell Precursor Acute Lymphoblastic Leukemia. Genomics. Hemasphere.

[14] Pui CH, Nichols KE, Yang JJ (2019). Somatic and germline genomics in paediatric acute lymphoblastic leukaemia. Nat Rev Clin Oncol.

[15] Coccaro Nicoletta, Anelli Luisa, Zagaria Antonella, Specchia Giorgina, Albano Francesco (2019). Next-Generation Sequencing in Acute Lymphoblastic Leukemia. International Journal of Molecular Sciences.

[16] Tran Anh Nhi, Taylan Fulya, Zachariadis Vasilios, Ivanov Öfverholm Ingegerd, Lindstrand Anna, Vezzi Francesco, Lötstedt Britta, Nordenskjöld Magnus, Nordgren Ann, Nilsson Daniel, Barbany Gisela (2018). High-resolution detection of chromosomal rearrangements in leukemias through mate pair whole genome sequencing. PLOS ONE.

[17] Lilljebjorn H (2016). Identification of ETV6-RUNX1-like and DUX4-rearranged subtypes in paediatric B-cell precursor acute lymphoblastic leukaemia. Nat Commun.

[18] Yasuda T (2016). Recurrent DUX4 fusions in B cell acute lymphoblastic leukemia of adolescents and young adults. Nature genetics.

[19] Marincevic-Zuniga Y (2017). Transcriptome sequencing in pediatric acute lymphoblastic leukemia identifies fusion genes associated with distinct DNA methylation profiles. Journal of hematology & oncology.

[20] Biondi A (2012). Imatinib after induction for treatment of children and adolescents with Philadelphia-chromosome-positive acute lymphoblastic leukaemia (EsPhALL): a randomised, open-label, intergroup study. The Lancet. Oncology.

[21] Nordlund J (2015). DNA methylation-based subtype prediction for pediatric acute lymphoblastic leukemia. Clinical epigenetics.

[22] Liu YF (2016). Genomic Profiling of Adult and Pediatric B-cell Acute Lymphoblastic Leukemia. EBioMedicine.

[23] Zhang J (2016). Deregulation of DUX4 and ERG in acute lymphoblastic leukemia. Nature genetics.

[24] Gunnarsson R (2018). Mutation, methylation, and gene expression profiles in dup(1q)-positive pediatric B-cell precursor acute lymphoblastic leukemia. Leukemia.

[25] Moorman AV (2014). A novel integrated cytogenetic and genomic classification refines risk stratification in pediatric acute lymphoblastic leukemia. Blood.

[26] Schwab CJ (2013). Genes commonly deleted in childhood B-cell precursor acute lymphoblastic leukemia: association with cytogenetics and clinical features. Haematologica.

[27] Kawazu M (2017). Integrative analysis of genomic alterations in triple-negative breast cancer in association with homologous recombination deficiency. PLoS genetics.

[28] Greer SU (2017). Linked read sequencing resolves complex genomic rearrangements in gastric cancer metastases. Genome medicine.

[29] Viswanathan SR (2018). Structural Alterations Driving Castration-Resistant Prostate Cancer Revealed by Linked-Read Genome Sequencing. Cell.

[30] Garcia S (2017). Linked-Read Sequencing for Molecular Cytogenetics. J Mol Diagn.

[31] Zhou B (2019). Haplotype-resolved and integrated genome analysis of the cancer cell line HepG2. Nucleic Acids Res.

[32] Clapp J (2007). Evolutionary conservation of a coding function for D4Z4, the tandem DNA repeat mutated in facioscapulohumeral muscular dystrophy. American journal of human genetics.

[33] Eisfeldt J (2019). Comprehensive structural variation genome map of individuals carrying complex chromosomal rearrangements. PLoS genetics.

[34] Ho SS, Urban AE, Mills RE (2019). Structural variation in the sequencing era. Nat Rev Genet.

[35] Xu, J. *et al*. An Integrated Framework for Genome Analysis Reveals Numerous Previously Unrecognizable Structural Variants in Leukemia Patients’ Samples. 563270, https://doi.org/10.1101/563270%JbioRxiv (2019).

[36] Janeway KA, Place AE, Kieran MW, Harris MH (2013). Future of clinical genomics in pediatric oncology. Journal of clinical oncology: official journal of the American Society of Clinical Oncology.

[37] Grobner SN (2018). The landscape of genomic alterations across childhood cancers. Nature.

[38] Milani L (2010). DNA methylation for subtype classification and prediction of treatment outcome in patients with childhood acute lymphoblastic leukemia. Blood.

[39] Paulsson K, Johansson B (2009). High hyperdiploid childhood acute lymphoblastic leukemia. Genes, chromosomes & cancer.

[40] Abyzov A, Urban AE, Snyder M, Gerstein M (2011). CNVnator: an approach to discover, genotype, and characterize typical and atypical CNVs from family and population genome sequencing. Genome research.

[41] Obenchain V (2014). VariantAnnotation: a Bioconductor package for exploration and annotation of genetic variants. Bioinformatics.

[42] Hiller B, Bradtke J, Balz H, Rieder H (2005). CyDAS: a cytogenetic data analysis system. Bioinformatics.

[43] Nicorici DS (2014). FusionCatcher - a tool for finding somatic fusion genes in paired-end RNA-sequencing data. bioRxiv..

[44] Nordlund J (2013). Genome-wide signatures of differential DNA methylation in pediatric acute lymphoblastic leukemia. Genome biology.

[45] Marzouka NA (2016). CopyNumber450kCancer: baseline correction for accurate copy number calling from the 450k methylation array. Bioinformatics.

[46] Rasmussen M (2011). Allele-specific copy number analysis of tumor samples with aneuploidy and tumor heterogeneity. Genome biology.

