# Supplementary material for: Refined detection and phasing of structural aberrations in pediatric acute lymphoblastic leukemia by linked-read whole-genome sequencing
Source: Sci Rep. 2020 Feb 13;10:2512. doi: 10.1038/s41598-020-59214-w (PMC7018692; doi:10.1038/s41598-020-59214-w)

**Refined detection and phasing of structural aberrations in pediatric acute lymphoblastic leukemia by linked-read whole-genome sequencing**

Jessica Nordlund^1^*, Yanara Marincevic-Zuniga^1^, Lucia Cavelier^2^, Amanda Raine^1^, Tom Martin^1^, Anders Lundmark^1^, Jonas Abrahamsson^3^, Ulrika Norén-Nyström^4^, Gudmar Lönnerholm^5^, Ann-Christine Syvänen^1^

^1^Department of Medical Sciences, Molecular Medicine and Science for Life Laboratory, Uppsala University, Sweden

^2^Department of Immunology, Genetics and Pathology and Science for Life Laboratory, Uppsala University, Sweden

^3^Department of Pediatrics, Institution for Clinical Sciences, Sahlgrenska Academy, Gothenburg University, Gothenburg, Sweden

^4^Department of Clinical Sciences and Pediatrics, University of Umeå, Sweden

^5^Department of Women’s and Children’s Health, Pediatric Oncology, Uppsala University, Sweden

**Figure S1**. Effect of genomic DNA fragment size on phasing and detection of fusion genes. The weighted average DNA size estimated by the 10x Genomics Long Ranger software is plotted against (A) the size of the largest phase block and (B) the median size (N50) of the phase block. High molecular weight (HMW) DNA (red dot), DNA extracted with a column-based method (standard, green triangles), and whole-genome amplified DNA (WGA, blue square) are indicated in the plots. HMW DNA yielded phased blocks that spanned 18 Mb of DNA, whilst standard column-based DNA that had undergone repeated freeze-thawing yielded shorter phased blocks ranging from 1-14 Mb. The sizes of the longest and the N50 phase blocks correlated with the average size of the input DNA estimated by the Long Ranger software (Supplementary Table 3). WGA DNA gave poor results. (C-D) Heatmaps of overlapping linked reads supporting inter-chromosomal translocations are plotted in orange (10x Genomics Loupe software). (C) HMW DNA extracted from fresh frozen cells from patient ALL_402 harboring t(9;22) and (D) standard column-based DNA extraction from the same patient. The expected breakpoints in the fusion genes were identified in both HMW standard column DNA extractions, although .the cumulative number of common barcodes shared between the two loci of each translocation resulted in stronger signal intensities in the HMW DNA (26,316 fluorescence units in C) compared to the standard column-based DNA sample (7,500 fluorescent units in D).

**
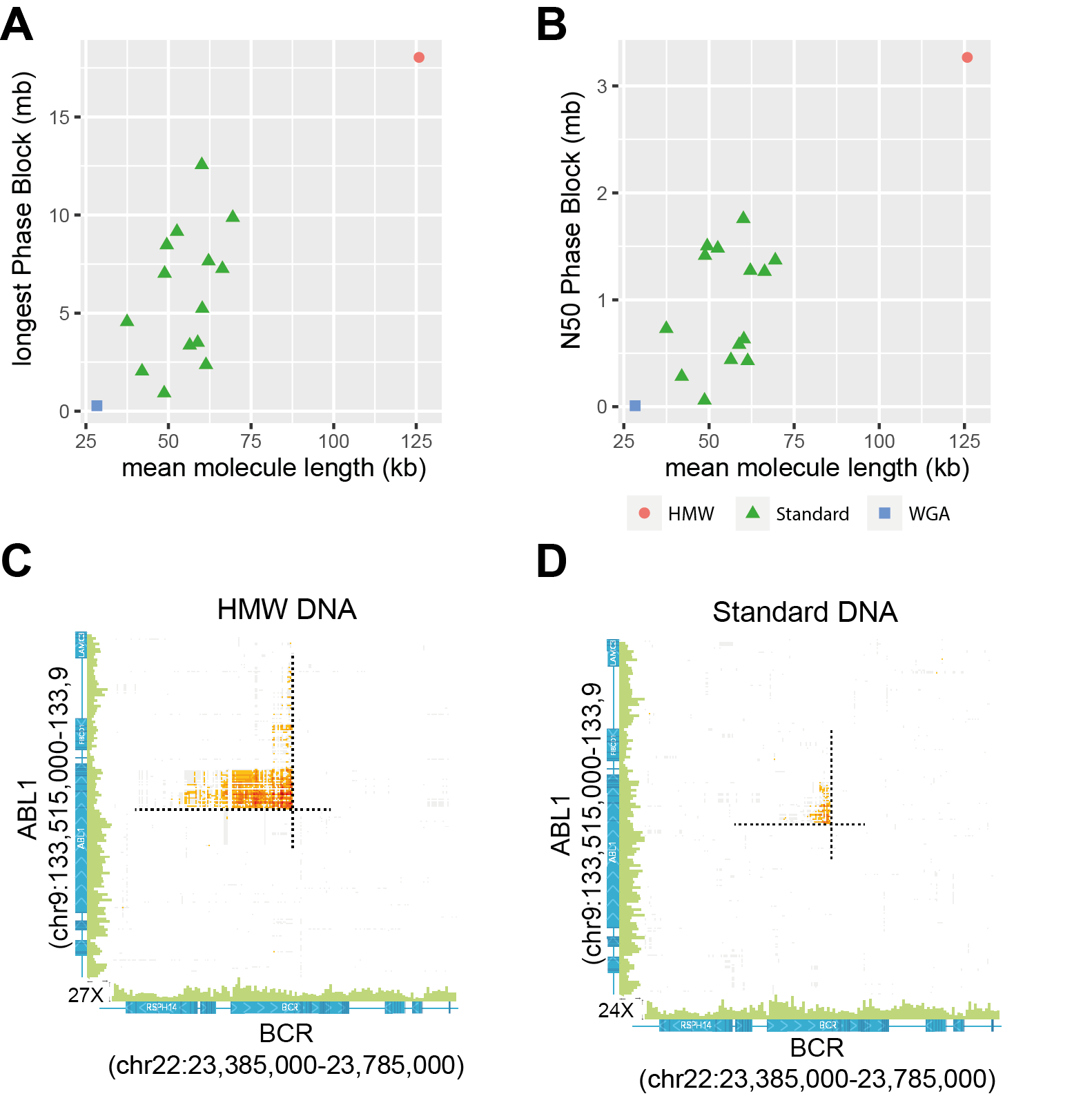
**

**Figure S2.** IGV snapshots depicting chromosomal copy number (upper panels red color) determined by linked-read WGS, 450k array or SNP array, and B-allele frequency (low panels blue color) determined by linked-read WGS or SNP arrays for three patients with HeH. The linked-read WGS and SNP array data was consistent for all patients (A-C). In patient ALL_47, a copy neutral LOH of chromosome 19 was observed in both the SNP array and linked-read WGS data.

**
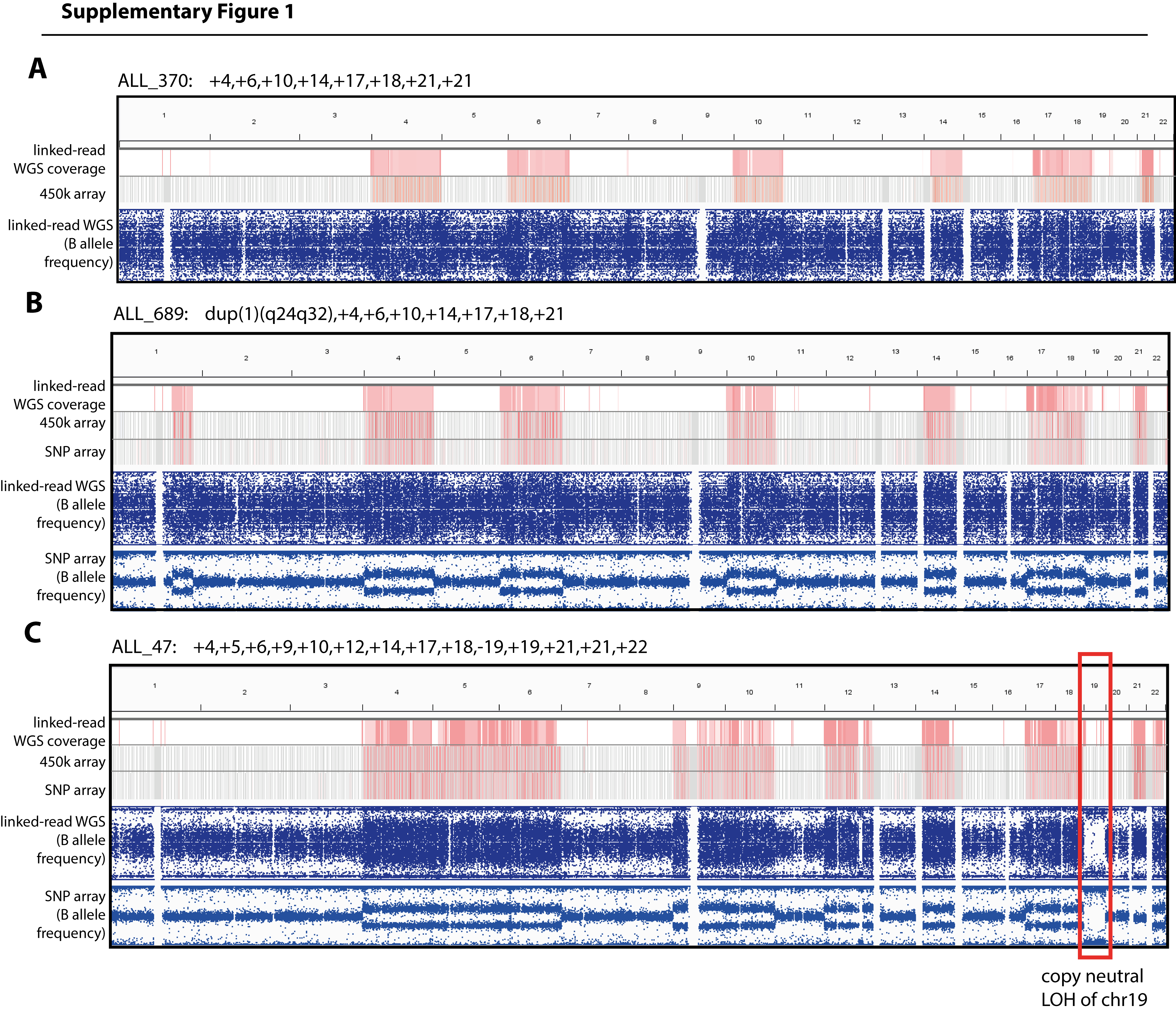
**

**Figure S3.** Linked-read WGS data for t(12;21)*ETV6-RUNX1* positive patient ALL_458. (A) Heatmap of overlapping linked-reads supporting a balanced inter-chromosomal translocation t(12;21) resulting in the *ETV6-RUNX1* fusion gene (Loupe software). (B) A 2.1 Mb deletion spanning the other allele of *ETV6* (not involved in the translocation). The enlarged panel to the right depicts the shared barcodes between the distant loci chr12:11,230,000 and chr12:13,330,000 owing to the 2.1Mb deletion as illustrated on top of the right most panel. (C) A schematic view of derivative chromosomes 12 and 21 including the t(12;21) translocation and the 2.1 Mb deletion on chr12. (D) IGV snapshot of chromosome 12 confirming the 2.1 Mb deletion spanning the *ETV6* locus in logR ratios from Illumina Infinium arrays (450k and SNP array) and coverage in 10kb windows from the linked-read WGS data.


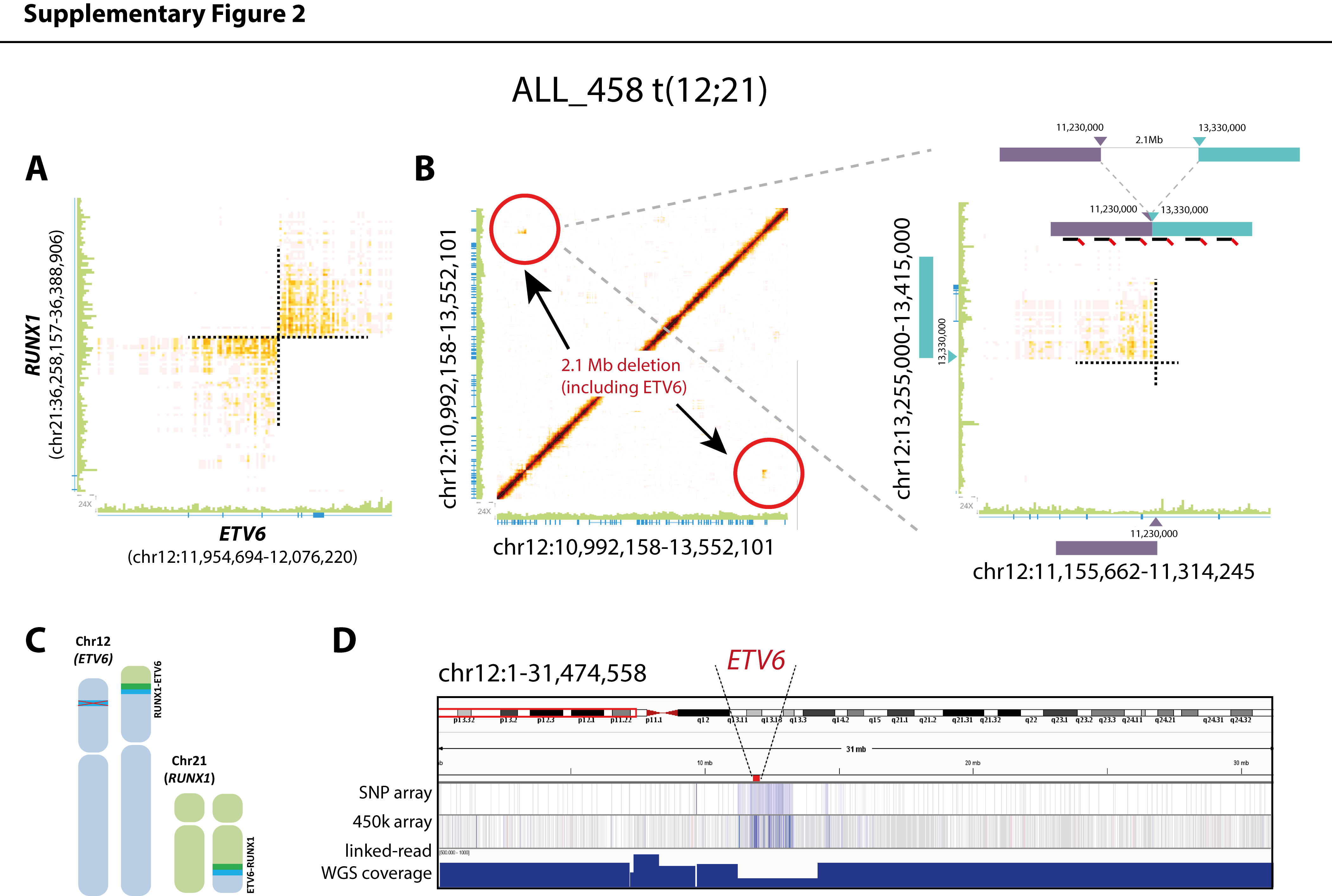


**Figure S4**. Snapshots from the Loupe browser depicting overlapping linked-reads supporting the series of inter-chromosomal translocations observed in ALL_386. (A) Translocation between chromosome 12p13.2 and 21q22.12, resulting in the expression of the *ETV6-RUNX1* fusion gene. (B) Translocation between chromosome 14q24.1 and 12p13.2 resulting in the *DCAF5-ETV6* fusion gene. (C) Translocation between chromosome 21q22.12 at the *RUNX1* locus and chromosome 2q33.1 (D) Translocation between chromosome 2q37.3 and chromosome 14q24.1 at the *DCAF5* locus (E) 45 Mb deletion on chromosome 2q33.1-q37.3. (F) A translocation of chromosome 3q21.3 to 12q24.11 resulting in derivative chromosome 12. (G) A translocation of chromosome 12q24.12/12q24.13 to 3p21.31 resulting in derivative chromosome 3* (see panel H as 3p21.31 is moved to the q arm of chromosome 3). (H) 650 kb insertion-inversion from chromosome 3p21.31 to chromosome 3q21.2. (I) 6.5 Mb deletion of chromosome 3p21.2-p21.31.

**
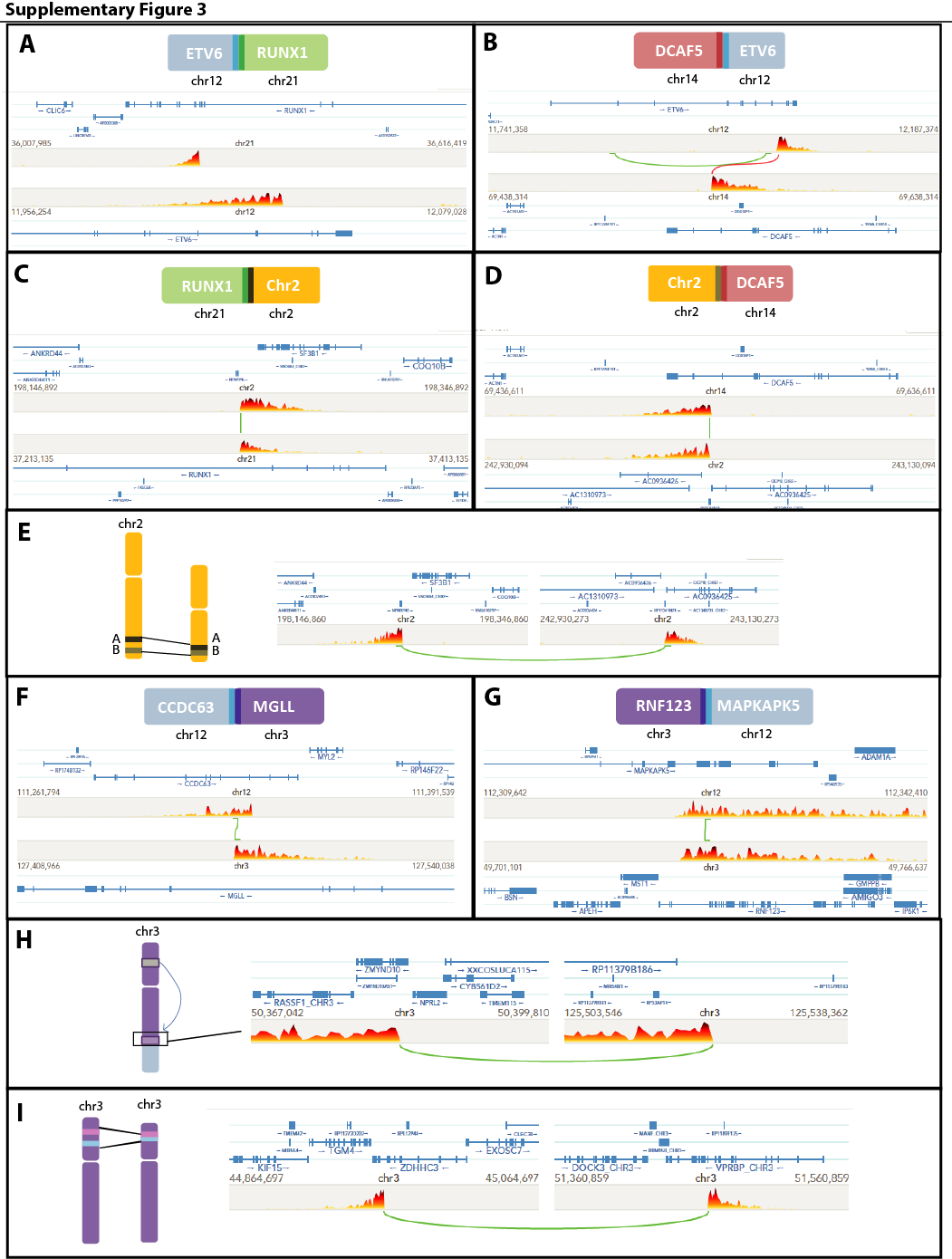
**

**Figure S5**. Derivative chromosomes in ALL_386. (A) Chromosome painting performed at diagnosis. Translocations confirmed by linked-read WGS are outlined in white and underlined text (B) Derivative chromosomes as determined by linked-read WGS. The ideograms were drawn to scale using the CyDAS software.


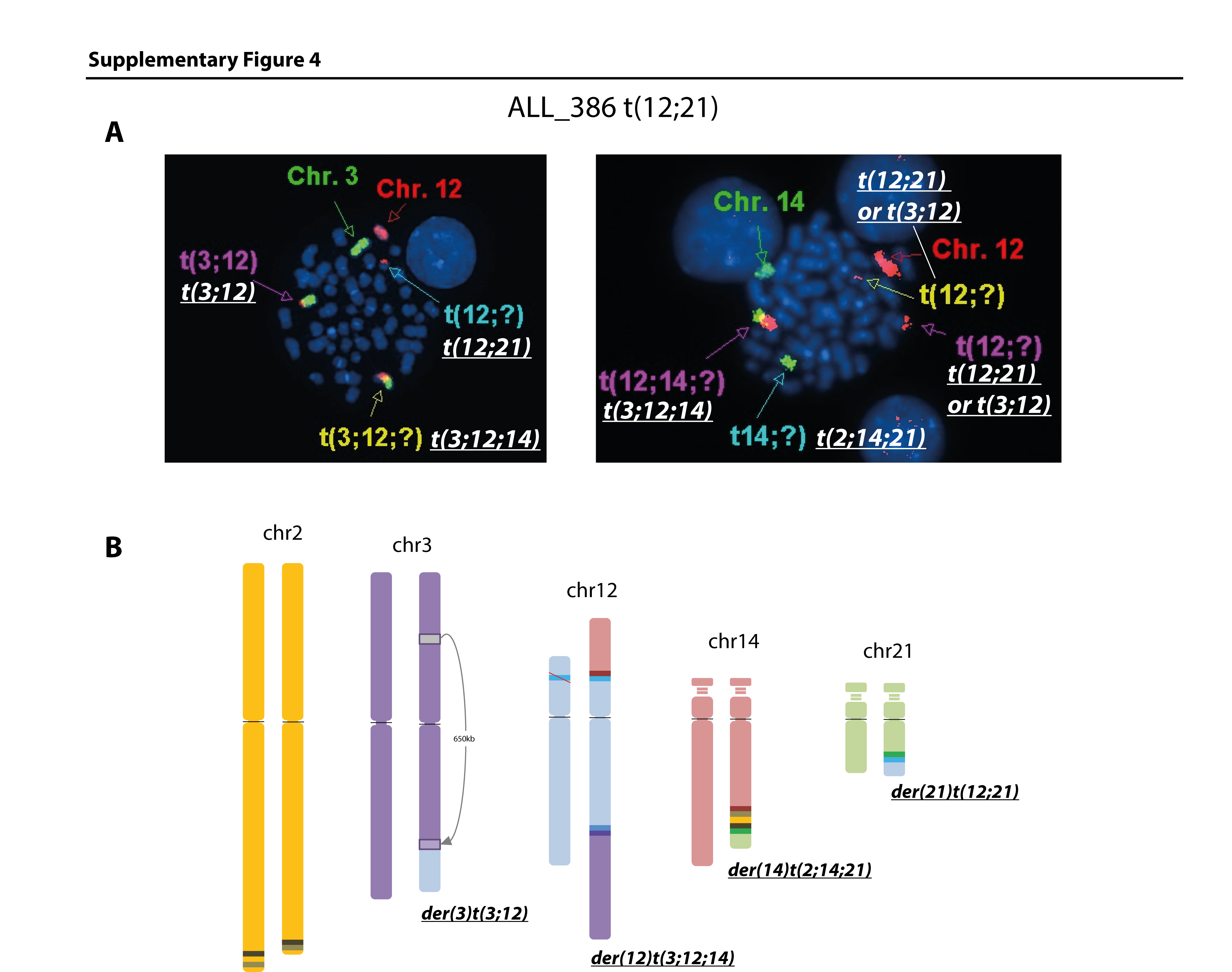


**Figure S6**. Snapshots from the Loupe browser depicting overlapping linked-reads supporting the series of interchromsomal translocations in ALL_402. (A) Unbalanced translocation resulting in the expected canonical t(9;22)*BCR-ABL1* based on karyotype for this patient. (B) Unbalanced translocation between chromosome 5q31.2 and 9q34.12 resulting in the expression of two truncated transcripts, *SIL1* and *ABL1,* respectively*.* (C) Unbalanced translocation between chromosome 1p36.33 and 5q31.2 resulting in the expression of *LINC01128-SIL1*. (D) Unbalanced translocation between chromosome 1p36.33 and chromosome 9q34.13. (E) Unbalanced translocation between chromosome 9q34.13 and chromosome 22q11.23 resulting in the expression of *PRRC2B-BCR.* (F) The same translocations as in panels A and E, zoomed out, showing the two translocations with the *BCR* locus on chromosome 22q11.23 with the *ABL1* and *PRRC2B* genes on chromosome 9p34.12-9p34.13.


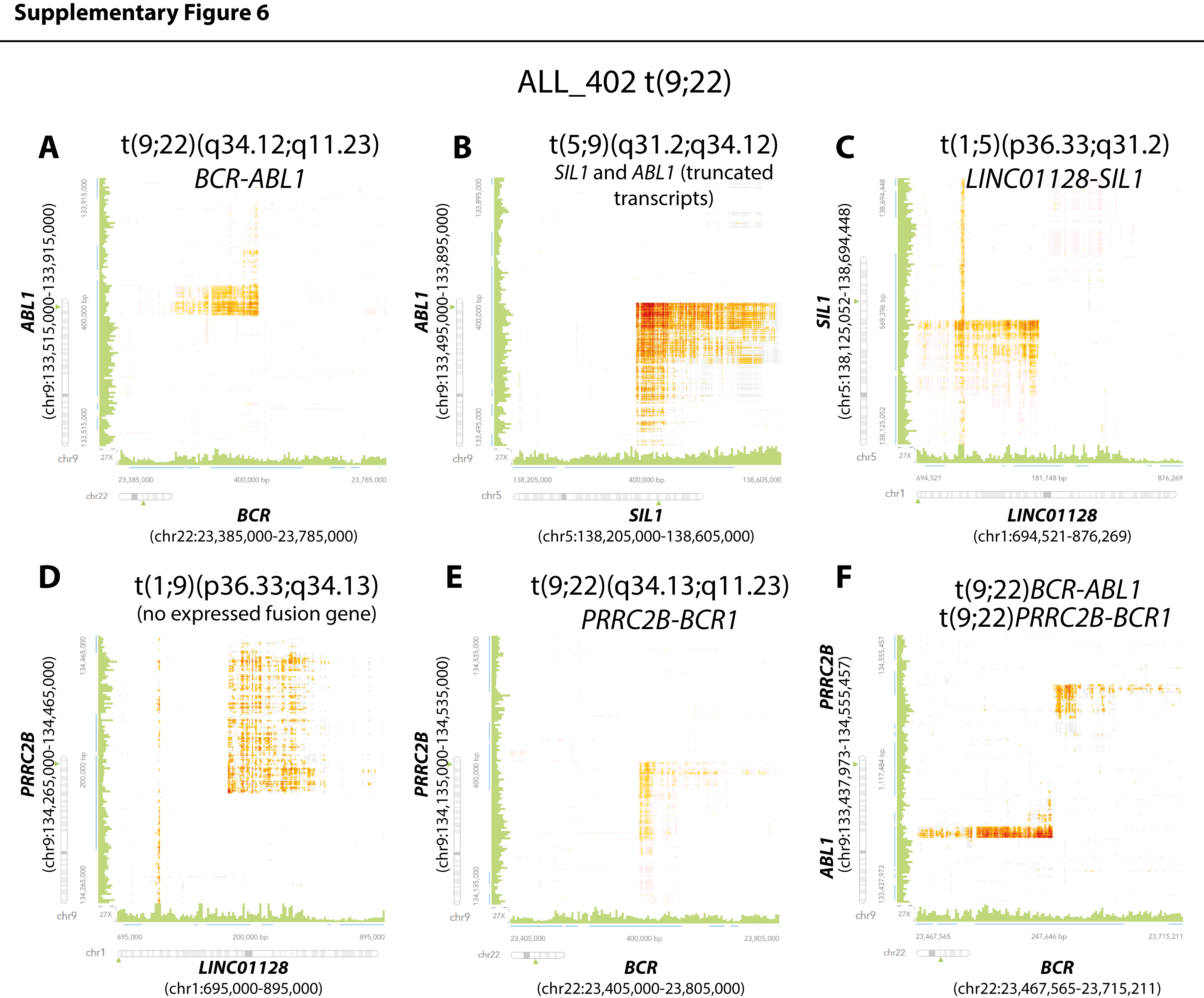


**Figure S7**. Circos plots for two patients with *DUX4-IGH*. The chromosomes and bands are plotted on the outermost track, followed by the logR ratios from Infinium arrays, linked-read WGS coverage calculated in 10kb bins, and copy number aberration calls obtained using the CNVnator software are indicated in blue for deletion and red for amplification. Fusion genes detected by RNA-sequencing are indicated in the interior of the plot. (A) The only large-scale aberration detected in ALL_390 is a deletion of chromosome 6q. (B) No large-scale aberrations were detected in ALL_501.

**
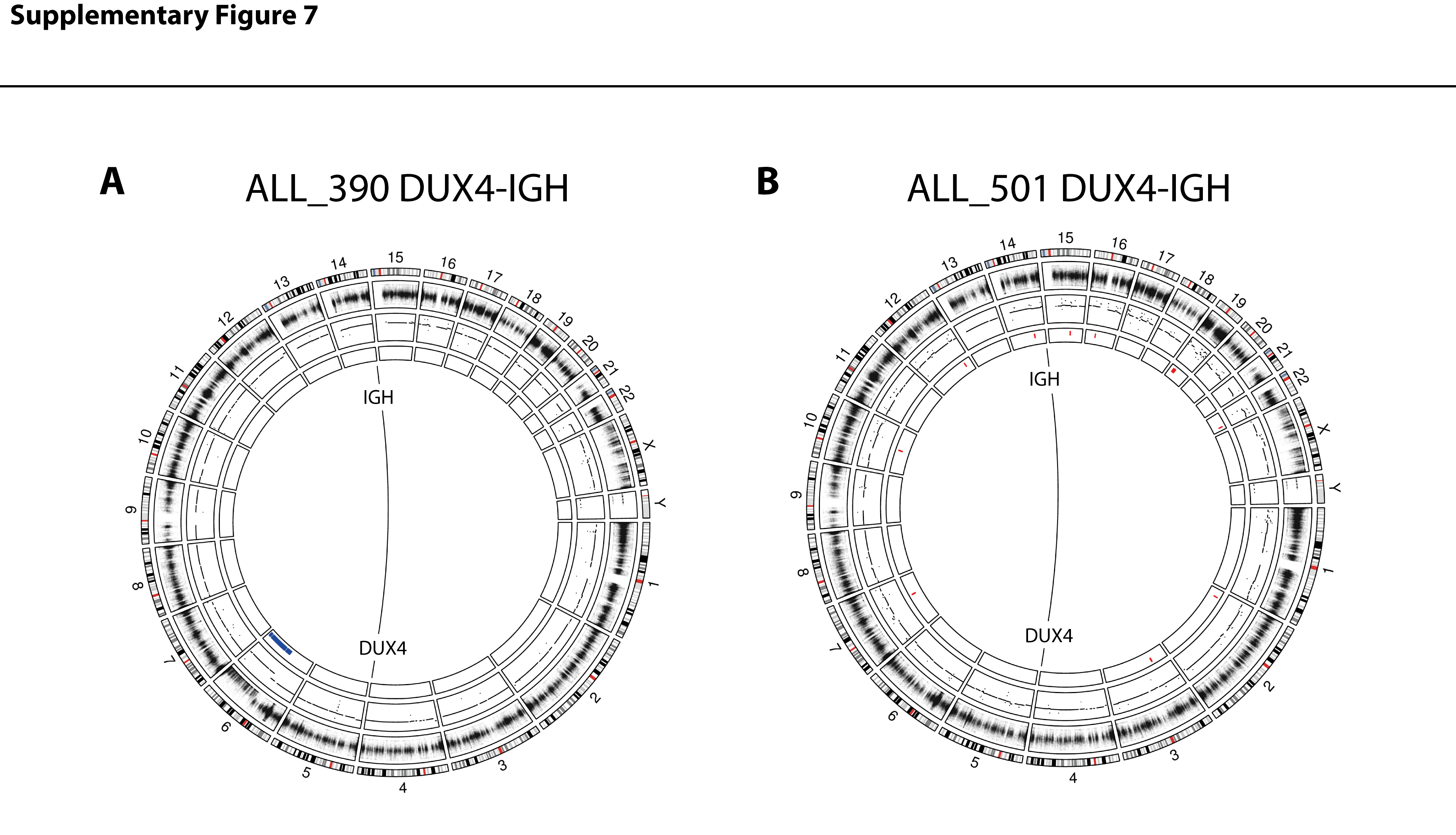
**

**Figure S8**. (A) Validation of small *ERG* deletion in ALL_390 by Sanger sequencing. The Sanger sequencing traces are marked in red in the two lower tracks and represent two different primer combinations (F1+R1 and F2+R2). The red square highlights the obtained Sanger sequences that span a 9.3kb deletion in *ERG*, including exon 1 of *ERG* variant NM_182918.3. (B-C) Snapshot from IGV of reads supporting the insertion of *DUX4* into the *IGH* locus. (B) Linked-read WGS reads are shown in the upper panels (highlighted with a red box) and RNA-seq reads are shown in the lower panels for ALL_390. (C) Only RNA-seq reads are shown for ALL_501 as no linked-read WGS reads supported the fusion due to the low 10x sequencing depth in this library.


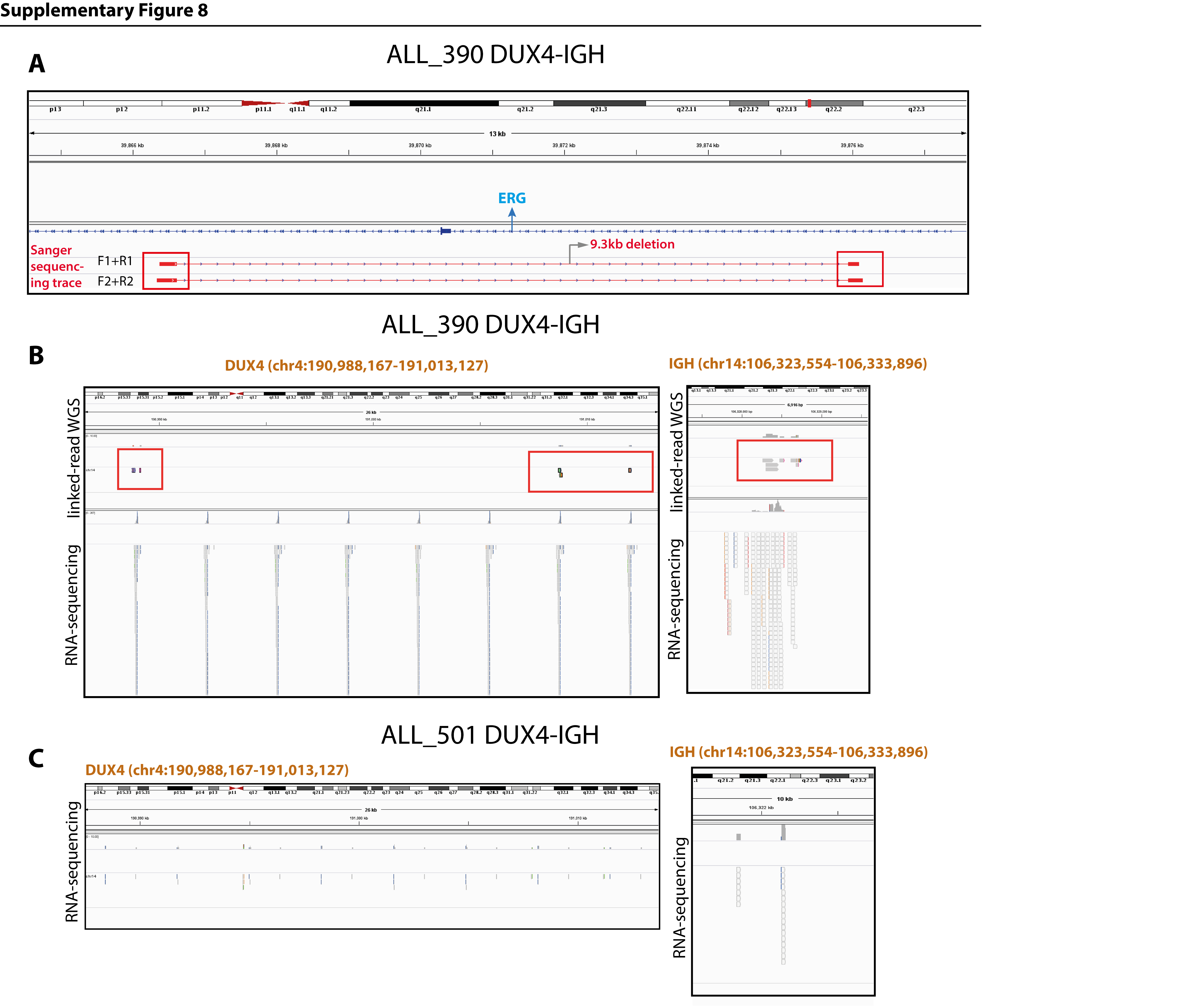


**Figure S9**. Circos plots for two patients with *ZNF384-*rearrangements. The chromosomes and bands are plotted on the outermost track, followed by the logR ratios from Infinium arrays, linked-read WGS coverage calculated in 10kb bins, and copy number aberration calls obtained using the CNVnator software are indicated in blue for deletions and red for gains. Fusion genes detected by RNA-sequencing are indicated in the interior of the plot. (A) Circos plot for ALL_604 depicting the translocation resulting in the *TCF3-ZNF384* fusion gene. (B) An IGV snapshot depicting the deletions on chromosome 6q16.2-q22.33and 7q21.3-q36.3 in the logR ratios from Infinium arrays and the 10kb binned coverage plot of the linked-read WGS data for ALL_604. (C) Circos plot for ALL_613 depicting the *EP300-ZNF384* fusion gene, amplification of the entire chromosome 1q arm and a deletion of chromosome 16q21-q24.3.

**
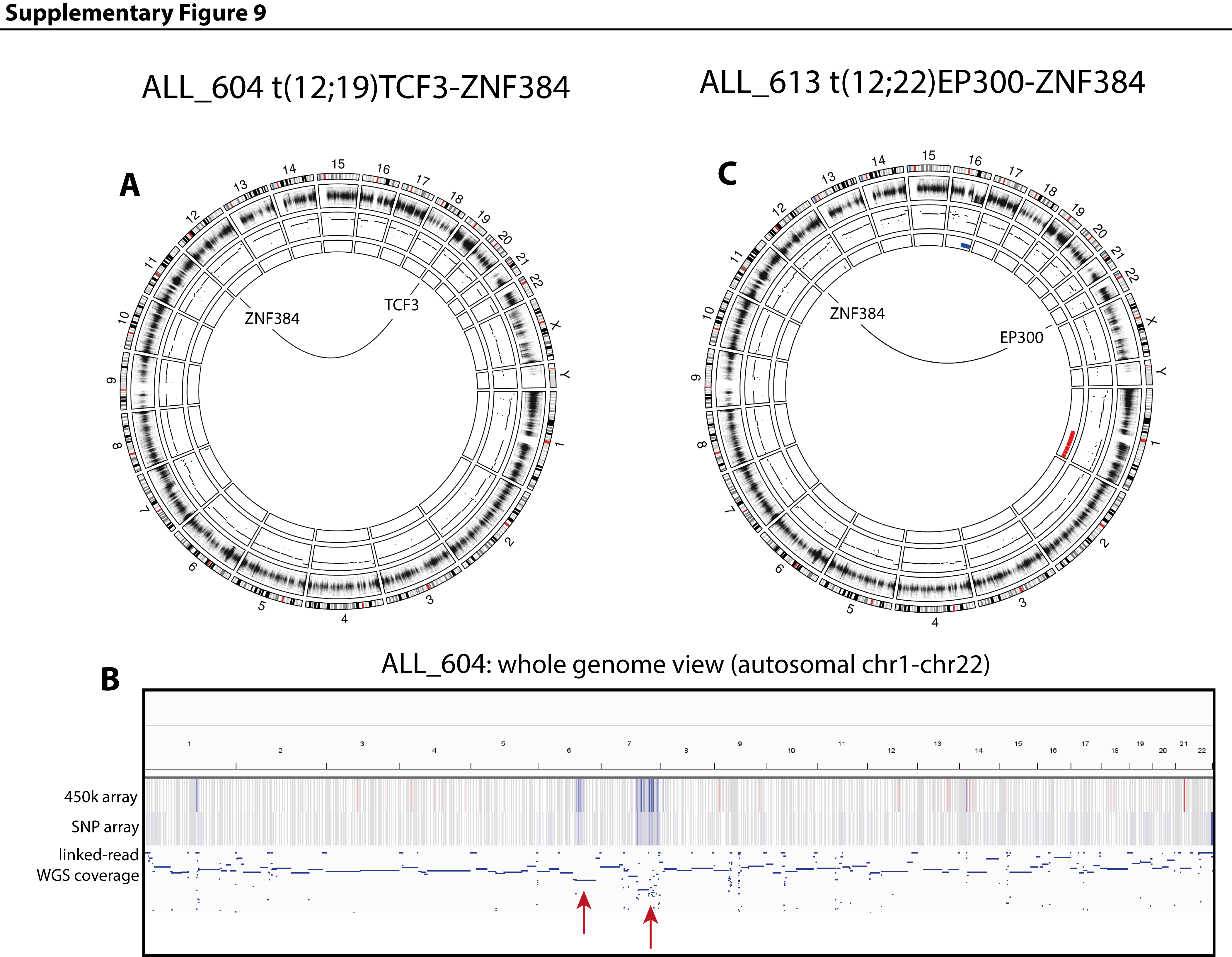
**

**Figure S10**. Genomic rearrangements in ALL_707. (A) Circos plot depicting the genome-wide copy number in ALL_707. The chromosomes and bands are plotted on the outermost track, followed by the logR ratios from Infinium arrays, linked-read WGS coverage calculated in 10kb bins, and copy number aberration calls obtained using the CNVnator software are indicated in blue for deletions and red for amplifications. Fusion genes detected by RNA-sequencing are indicated in the interior of the plot. (B) Snapshot from the Loupe browser depicting overlapping linked-reads supporting the translocation (7;9)(q11;p13) resulting in a derivative chromosome 9 harboring the *PAX5*-*ELN* fusion gene. (C) IGV snapshot of the genome-wide copy number determined by linked-read WGS coverage in 10kb windows and in Infinium arrays (logR ratios). Deletions are indicated in blue. (D) Zoomed-in view showing the breakpoint of the chromosome 9p deletion in the *PAX5* gene.


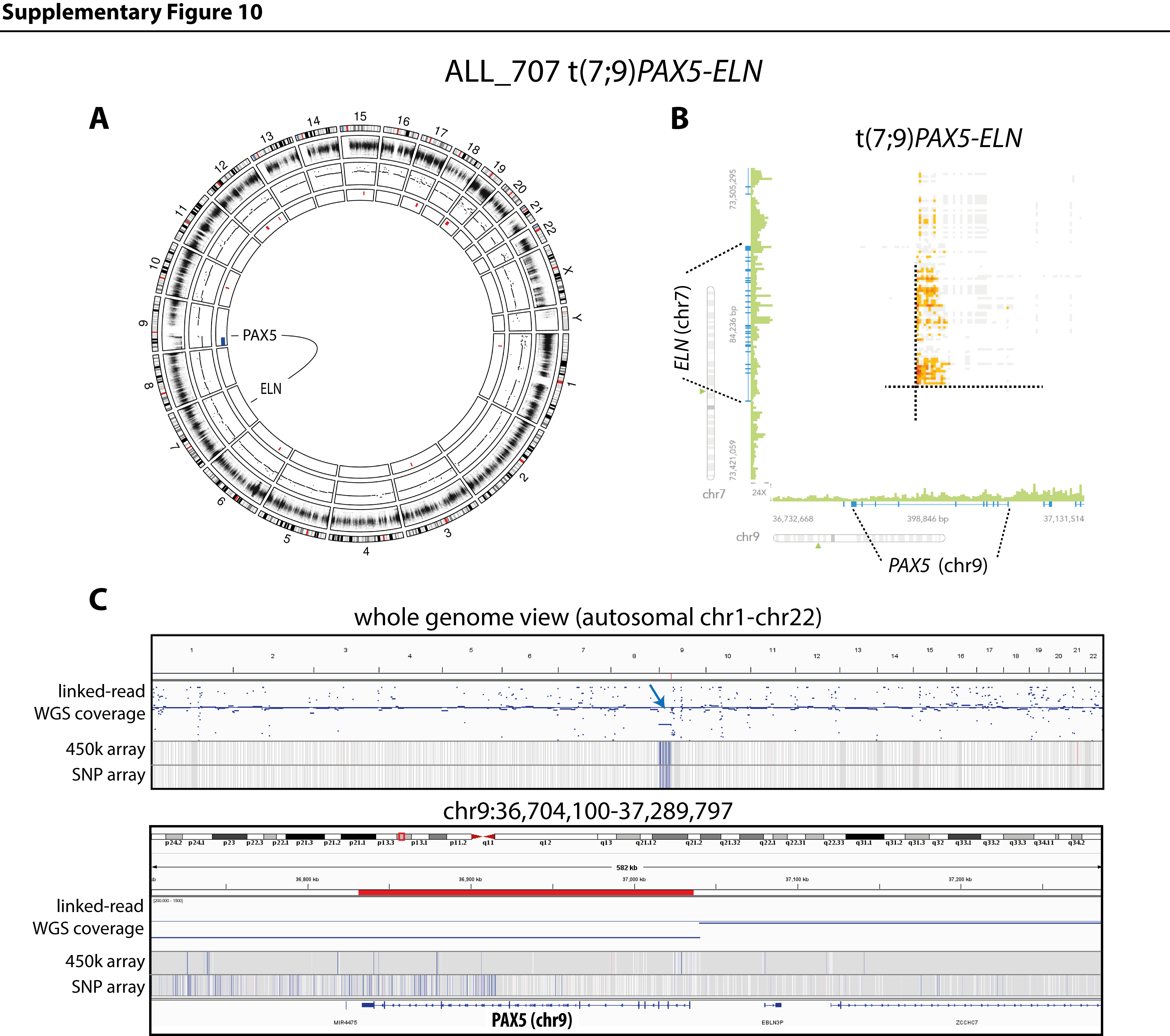


**Figure S11**. Genomic rearrangements in ALL_559. (A) Circos plot depicting the genome-wide copy number in ALL_559. The chromosomes and bands are plotted on the outermost track, followed by the logR ratios from Infinium arrays, linked-read WGS coverage calculated in 10kb bins, and copy number aberration calls obtained using the CNVnator software are indicated in blue for deletions and red for amplifications. Fusion genes detected by RNA-sequencing are indicated in the interior of the plot. (B) Heatmap from the Loupe browser depicting overlapping linked-reads supporting a large homozygous deletion spanning *CDKN2A/B* on chromosome 9p21.


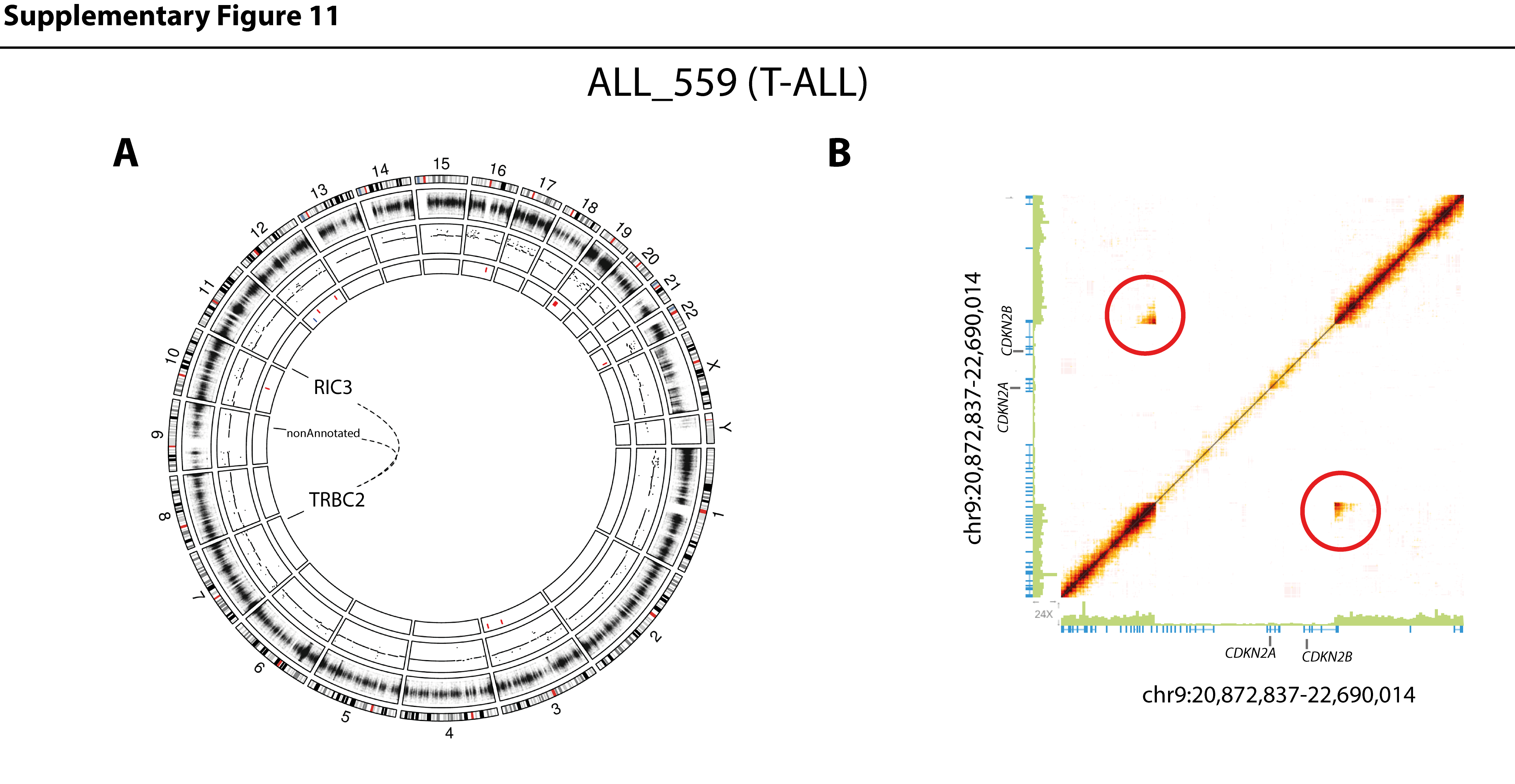


**Figure S12**. Focal deletions of a panel of diagnostically relevant genes for ALL. Each patient and library is indicated in a column and the genes examined for deletions are listed in rows. Deletions are color-coded according to the key to the right of the plot.


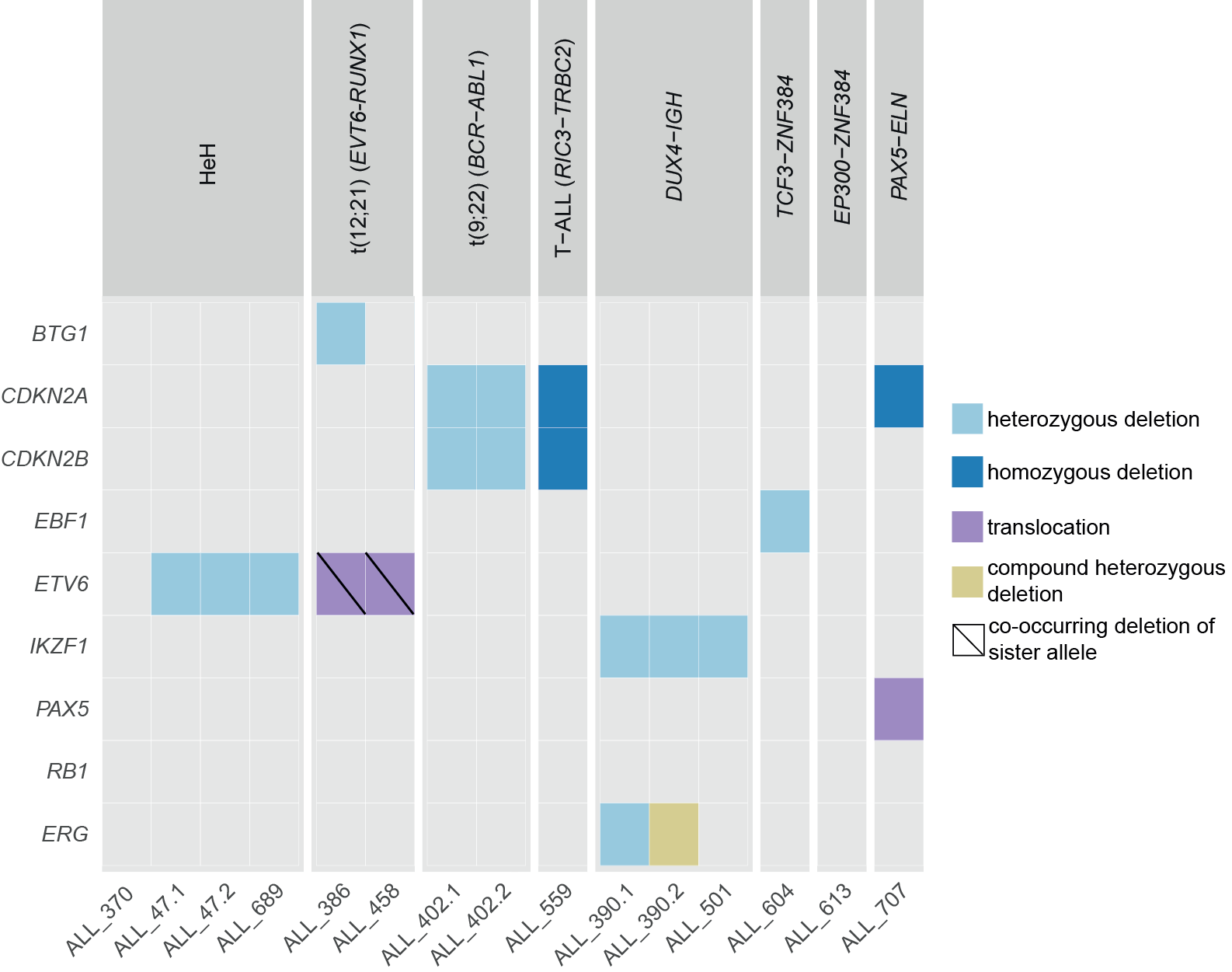


**Figure S13**. Focal deletions in patients with *DUX4-IGH*. (A-B) IGV snapshot of chromosome 7 at the *IKZF1* locus. The coverage in 10kb bins is plotted in the upper track, followed by the logR ratios from Infinium arrays, validating the linked-read WGS deletion calls. (C-D) Snapshot from the Loupe browser showing the linked-reads spanning the deletions in *IKZF1* on each of the two haplotypes. (E-H) Snapshot from the Loupe browser spanning the *IKZF1* locus showing overlapping linked-reads (E-F) and barcode coverage (G-H) supporting the deletion calls. ALL_501 was only sequenced to 10x coverage (GemCode) and the deletion was not as clearly resolved as in ALL_390 that was sequenced to 30x (Chromium).


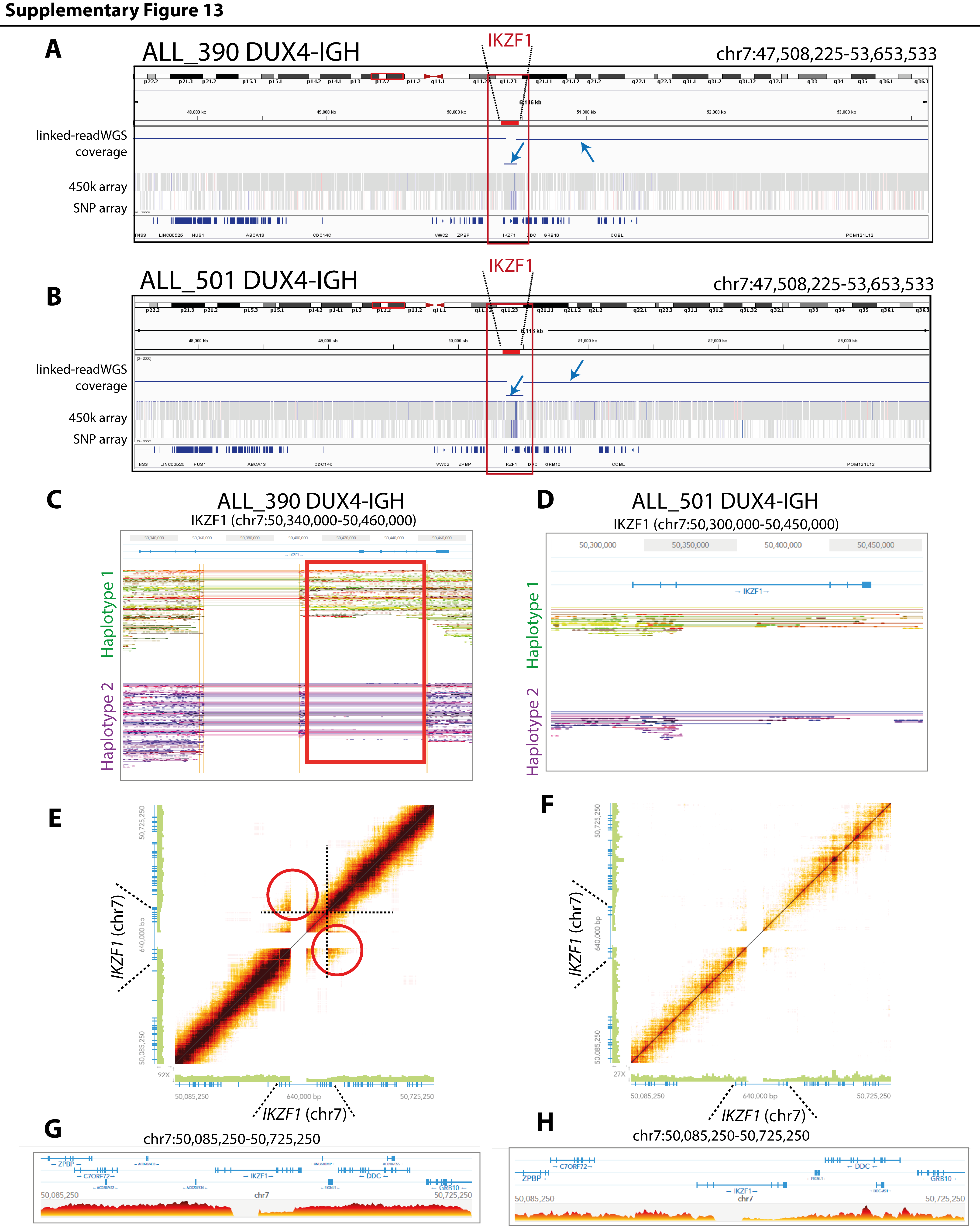

Supplement: Supplementary file 1 — Supplementary Figures S1-S13. [file 41598_2020_59214_MOESM1_ESM.docx]
